# What variables should be considered in allocating Primary health care Pharmaceutical budgets to districts in Uganda?

**DOI:** 10.1186/s40545-014-0023-1

**Published:** 2015-02-10

**Authors:** Paschal N Mujasi, Jaume Puig-Junoy

**Affiliations:** Department of Economics and Business, Universitat Pompeu Fabra, Barcelona School of Management, Balmes 132, 08001 Barcelona, Spain; Department of Economics and Business, Centre for Research in Health Economics (CRES), Universitat Pompeu Fabra, Ramon Trias Fargas 25-27, 08005 Barcelona, Spain

**Keywords:** Budget allocation, Econometric analysis, Needs-based formula, Pharmaceutical expenditure, Primary Health care, Uganda

## Abstract

**Objectives:**

A key policy question for the government of Uganda is how to equitably allocate primary health care pharmaceutical budgets to districts. This paper seeks to identify variables influencing current primary health care pharmaceutical expenditure and their usefulness in allocating prospective pharmaceutical budgets to districts.

**Methods:**

This was a cross sectional, retrospective observational study using secondary administrative data. We collected data on the value of pharmaceuticals procured by primary health care facilities in each district from National Medical Stores for the financial year 2011/2012. The dependent variable was expressed as per capita district pharmaceutical expenditure. By reviewing literature we identified 26 potential explanatory variables. They include supply, need and demand, and health system organization variables that may influence the demand and supply of health services and the corresponding pharmaceutical expenditure. We collected secondary data for these variables for all the districts in Uganda (n = 112). We performed econometric analysis to estimate parameters of various regression models.

**Results:**

There is a significant correlation between per capita district pharmaceutical expenditure and total district population, rural poverty, access to drinking water and outpatient department (OPD) per capita utilisation.(P < 0.01). The percentage of health centre IIIs (HC III) among each district’s health facilities is significantly correlated with per capita pharmaceutical expenditure (P < 0.05). OPD per capita utilisation has a relatively strong correlation with per capita pharmaceutical expenditure (r = 0.498); all the other significant factors are weakly correlated with per capita pharmaceutical expenditure (r < 0.5).

From several iterations of an initially developed model, the proposed final model for explaining per capita pharmaceutical expenditure explains about 53% of the variation in pharmaceutical expenditure among districts in Uganda (Adjusted R^2^ = 0.528). All variables in the model are significant (p < 0.01).

**Conclusions:**

From evaluation of the various models, proposed variables to consider in allocating prospective primary health care pharmaceutical budgets to districts in Uganda are: district outpatient department attendance per capita, total district population, total number of government health facilities in the district and the district human poverty index.

## Introduction

Government funding for essential medicines in Uganda is through National Medical Stores VOTE 116, an account established by the government to effectively and efficiently supply essential medicines and health supplies to public sector health facilities in the country. The National Medical Stores (NMS), a Ministry of Health (MOH) parastatal in charge of procurement, storage and distribution of health commodities manages the funds. Health facilities in the various districts are allocated budgets from these funds and they procure pharmaceuticals from the NMS against their allocated budget [1].

A key policy question for the government is how to equitably allocate the pharmaceutical budget to the various districts and health facilities in the districts. The current formula used by NMS to allocate the essential medicines and health supplies budget is loosely based on a district’s population size, mortality indicators and live births. This rough capitation formula with population-based distribution can be improved with addition of corrective factors. This creates the need to determine variables that influence pharmaceutical expenditure in the various districts in Uganda. This will help to identify corrective factors that can be used to improve the current capitation formula for budget allocation; or that can be used to develop alternative criteria for pharmaceutical budget allocation to districts.

Two popular approaches for pharmaceutical budget allocation are the use of historical costs, and the use of capitation based formulae that take into account the targeted population. There are a number of problems with using historical costs as a basis for budget setting. Firstly, there is no guarantee that the existing distribution is efficient or equitable. Secondly budgets set on the basis of historical costs may be subject to manipulation; health workers may have the incentive to increase their current prescribing costs in the hope of receiving larger budgets in the future [2]. Capitation formulae based on targeted population are an attempt to link pharmaceutical budgets to the needs of the targeted population. However, variations in pharmaceutical expenditure can still be observed in districts with similar populations. Such unexplained variations are liable to be interpreted as indicating inefficiency. If the main cause of the unexplained variations is idiosyncratic prescribing by health workers, the introduction of capitation based budgets would gradually move districts with high pharmaceutical expenditure towards the national average. It is also possible, though, that the unexplained variation in pharmaceutical expenditure is the result of differences in the clinical characteristics or socio-economic conditions of the district populations. If this is the case, rough capitation-based budgets may lead to unfair distribution of resources. It is therefore important to explore the relationship between pharmaceutical expenditure on one hand, and socioeconomic and demographic features on the other, to enable refinement of capitation based allocation formulae.

Studies in Spain, England and Italy have shown the following factors to be associated with pharmaceutical expenditure in primary health care (PHC) services at the health care area level: socio-demographic structure, morbidity of the population, variables associated with health care utilization [3-7] location and health system organizational factors [8] and the quality of prescribing by health workers [6]. These factors influence the demand and supply of health services and the related pharmaceutical expenditure. However, little research has been conducted in Uganda to validate these findings or to determine which variables affect pharmaceutical expenditure in PHC services. Determining such variables would help in refining the allocation criteria for pharmaceutical budgets to districts.

With the specific aim of aiding budget setting, Forster and Frost attempted to explain differences in prescribing rates and costs between family practitioner committee areas in England and Wales based on regression models [9]. They concluded that 60% of the variation in prescribing costs per patient could be explained by differences in the age/sex distribution of the population, standardised mortality rates and the supply of General Practitioners (GPs) per head of population. Levels of deprivation (measured by the Jarman index) were also considered but were found to be unimportant. Similar results were obtained using the number of prescriptions per person rather than the cost per person as the dependent variable.

As part of a more general analysis of practice variation in primary care, Baker and Klein examined differences in GP prescribing rates across family health service areas (FHSAs) [10]. Using step wise regression analysis, they were able to explain 69% of the variation in prescribing rates. Explanatory variables found to be important were similar to those in Forster and Frost’s study [9]: standardized mortality ratios, the supply of GPs per capita and the proportion of the population aged over 65 years. An additional variable, the number of ancillary staff per practitioner was found to be significant. Again, Jarman index was not significant.

The aim of this paper is to identify variables explaining current primary health care pharmaceutical expenditure by districts in Uganda, and to assess the usefulness of these variables in allocating prospective pharmaceutical budgets to the districts. Using regression analysis, the paper examines various models to explain variations in per capita pharmaceutical expenditure at the district level in Uganda. The paper provides recommendations for a final model to be used for pharmaceutical budget allocation to the various districts.

## Methods

### Study design

This was a cross sectional, retrospective observational quantitative study using secondary administrative data.

### Sample

The sample comprised of all the 121 districts in Uganda in the FY 2011/2012.

### Data collection

We collected from MOH, data on the value of pharmaceuticals supplied by NMS to health facilities in each district excluding district, regional and national referral hospitals. The collected data was for a one year period corresponding to the financial year (FY) 2011/2012 (July 1, 2011-June 30, 2012). The data excluded budget lines for artmesinin based combination therapies (ACTs) for Malaria, antiretrovirals (ARVs), Tuberculosis medicines, reproductive and maternal health supplies, commodities for health emergencies and vaccines for immunizations. These were excluded because their funding, which is mainly provided by donors, is centralised and districts are not restricted to how much they can receive. In contrast, funding for essential medicines and health supplies (EMHS) is solely provided by government through the government budgeting process and districts are allocated prospective budgets which they are not expected to exceed. Given that once allocated the funding for essential medicines is not fungible between districts, ensuring optimum budget allocation to the districts is very important, hence the focus on this budget line.

From literature review [2-10], we identified 26 variables related to supply, need and demand, and health system organization that might influence the demand and supply of health services and the related pharmaceutical expenditure (explanatory variables). Data for these variables were obtained for all the districts in Uganda (n = 112) from MOH and Government of Uganda (GoU) data bases and from various administrative reports and publications. The variables are shown in Table 1. An Excel data base was established for the collected data.

**Table 1:** **Explanatory variables representing need and demand, supply and health system organization factors**

**Group of factors**	**Variables**	**Description**	**Data source**
Need and demand	POPTOT	Total projected district population,2012	Uganda National Bureau of Statistics
	PERCFEM	Percentage of Female district population, 2012	Uganda National Bureau of Statistics
	RURALPOV	Percentage of rural population below poverty line, 2005	National Household Survey, 2005
	DPT3COVER	Percentage of children fully immunized against Diphtheria, Pertusis & Tuberculosis (Indicator of Immunisation coverage)	MOH Annual Performance Report, 2011/2012
	OPDCAPITA	Outpatient attendance per capita	MOH Annual Performance Report, 2011/2012
	HDI	Human Development Index;	Uganda Human Development Report, 2007
	HPI	Human Poverty Index;	Uganda Human Development Report, 2007
	ACCESSWATER	Percentage of district population with access to Safe drinking Water	State of the Uganda Population Report;2008
	LATCOVERAGE	Latrine Coverage in Households:% of households with latrine	State of the Uganda Population Report;2008
	URBANISATION	Urbanization level; Percentage of district considered to be urban	National Census; 2002
	LABOURABSRATE	Labour Absorption Rate	National Census; 2002
	LITRATETotal	Total Literacy Rate in the district	State of the Uganda Population Report;2008
	LITRATEFemale	Female Literacy Rate	State of the Uganda Population Report;2008
	LITRATEMale	Male Literacy Rate	State of the Uganda Population Report;2008
Supply	RRHAVAIL	Availability of Regional Referral hospital in the district: Yes = 1No = 0	MOH Annual Performance Report, 2011/2012
	DISTAGE	Whether it’s a newly created district or not. = 1 if Yes; =0 if Not	MOH Annual Performance Report, 2011/2012
	DISTACCESS	Whether the district is characterized by MOH as hard to reach or not. =1 if Yes; =0 if Not	MOH Annual Performance Report, 2011/2012
	HFGOVTOT	Total Number of Government Health facilities in the district (excluding hospitals)	MOH Facility Inventory Report, 2012
	HOSPTOT	Total Number of Hospitals, both government and private, in the district	MOH Facility Inventory Report, 2012
	HFNGO	Total Number of Non Government Organisation (NGO) health facilities in the district	MOH Facility Inventory Report, 2012
Health system organisation	PERCHCII	Percentage of government health facilities that are HC II	MOH Facility Inventory Report, 2012
	PERCHCIII	Percentage of government health facilities that are HC III	MOH Facility Inventory Report, 2012
	PERCHCIV	Percentage of government health facilities that are HC IV	MOH Facility Inventory Report, 2012
	STAFFSTRENGTH	Percentage of approved staff posts filled	MOH Annual Performance Report, 2011/2012
	TA	Availability of donor funded Technical Assistance to health facilities in the district for Pharmaceutical Management:1 if Yes; =0 if Not	Securing Ugandan’s Right to Essential Medicines (SURE) Project Report
	ACCESS	Percentage of the district population that live within 5 km to a health facility	State of the Uganda Population Report;2008

We collected data for 26 potential explanatory variables but the goal was to identify a final allocation model with not more than five variables, based on an iteration of several models with various combinations of the 26 variables. Having a parsimonious model is important because for the allocation model to have practical application, it must be based on a few variables with readily available data.

### Data analysis

We exported the collected data that we had entered in Excel into SPSS. A key assumption was that the various variables were randomly distributed. This assumption is reasonable given the large number of districts involved in the study (n = 112).

We conducted statistical analysis using SPSS Version 16. The unit of analysis was the district. We performed the following analyses:

1. Univariant descriptive analysis to ascertain the shape of the distribution of each variable and to discover existence of outliers. We used summary statistics (maximum, minimum, mean and standard deviation) for this analysis.
2. Bivariant descriptive and inferential analysis to measure the association between the continuous variables and to compare means between groups of districts based on the dichotomic variables. We used Pearson’s correlation analysis and the equality of means test.
3. Econometric analysis using step wise multiple linear regression to estimate parameters of various regression models using Ordinary Least Squares (OLS) and hypothesis tests for the value coefficients.

### Variables

Primary health care pharmaceutical expenditure, the dependent variable was defined as per capita primary health care pharmaceutical expenditure (PHCPECapita): average value in Uganda Shillings (UGX) of pharmaceuticals supplied by NMS in one year, to health facilities in each district per district inhabitant based on projected 2012 district population.

Mean per capita pharmaceutical expenditure was 1134.7 UGX (~0.45 US $) and ranged from 280 (~0.11 US $) to 2800 UGX. (~1.11 U$).

The explanatory variables used in performing the multiple linear regression analysis to determine variations in PHC pharmaceutical expenditure among the districts comprised of four dichotomous and 22 continuous variables. Analysis of the shape of the distribution of the continuous variables using Shapiro Wilks W test indicated that all the variables were normally distributed. Table 2 shows a description of the variables.

**Table 2:** **Description of explanatory variables**

**Continuous variables**	**Minimum**	**Maximum**	**Mean**	**Standard deviation**
Total Population: POPTOT	54,000	1,723,300	304,774.64	227,539.57
Percentage Female: PERCFEM	40%	55%	50.6%	1.89
Percent rural population below poverty line: RURALPOV	7.7%	88.5%	39.4%	18.54
Immunisation Coverage: DPT3COVER	2.5%	100%	85.2%	18.24
Per Capita OPD utilisation: OPDCAPITA	0.3	3.4	1.18	0.54
Access to Safe drinking Water: ACCESSWATER	14.6%	97.6%	58.54	16.55
Latrine Coverage in Households: LATCOVERAGE	5%	97.6%	67.86	18.94
Human Development Index: HDI	0.216	0.660	0.531	0.0776
Human Poverty Index: HPI	9.6	65.3	30.75	9.86
Labour Absorption Rate: LABOURABSRATE	16.3%	70.7%	52.9%	9.34
Urbanisation Rate: URBANISATION	1.1%	100%	8.17	10.24
Literacy Rate-Total: LITRATETotal	11.6%	93.7%	64.52%	15.79
Literacy Rate-Female: LITRATEFemale	8.5%	92.2%	56.7%	16.84
Literacy Rate-Male: LITRATEMale	14.8%	95.4%	72.9%	15.95
Number of Government Health facilities: HFGOVTOT	8	88	25	15.22
Number of hospitals in district:HOSPTOT	0	28	1.36	2.81
Number of NGO health facilities: HFNGO	0	37	7.22	7.27
Percentage of facilities that are HC II: PERCHCII	19%	85%	56%	14.85
Percentage of facilities that are HC III: PERCHCIII	12%	75%	35.7%	13.784
Percentage of facilities that are HC IV: PERCHCIV	0%	21%	6%	4.405
Percentage of Approved posts filled: STAFFSTRENGTH	19%	86.6%	54.4%	14.0126
Percentage of population within 5 km to a Health Facility: ACCESS	43.7%	96.5%	69.7%	11.6607
**Dichotomic variables**	**Total N**	**Condition**	**Frequency**	**Percentage**
Availability of Regional Referral Hospital: RRHAVAIL	112	Yes	15	13.4%
		No	97	86.6%
Newly created district: DISTAGE	112	Yes	31	27.7%
		No	81	72.3%
Hard to reach district: DISTACCESS	112	Yes	25	22.3%
		No	87	77.7%
Availability for TA for Pharmaceutical Management: TA	112	Yes	45	40.2%
		No	67	59.8%

## Results

### Test of null hypothesis of no difference in per capita pharmaceutical expenditure between categories of districts

Before performing the regression analysis, we performed an independent samples *t*-test for the dichotomous variables to assess the null hypothesis that there is no difference in per capita pharmaceutical expenditure in each of the two groups for the dichotomous variables. The variables considered included: availability of regional referral hospital in the district (RRHAVAIL), whether the district was recently created or not (DISTAGE), whether the district is categorized by MOH as hard to reach or not (DISTACCESS) and whether external technical assistance for pharmaceutical management was available to the district (TA). The results are shown in Table 3. For all the variables considered, the null hypothesis (no difference in per capita pharmaceutical expenditure between districts) couldn’t be rejected (P > 0.05).

**Table 3:** **Comparison of the mean pharmaceutical expenditure per capita (′000) according to levels of dichotomic variables**

**Availability of RRH (RRHAVAIL)**	**Yes (n = 15)**	**Std Deviation**	**No (n = 97)**	**Std Deviation**	**Means difference**	**t**	**Sig. (2 tailed)**
	**1.1843**	**0.37685**	**1.1270**	**0.47372**	**0.05728**	**0.446**	**0.656**
Newly created district (DISTAGE)	Yes (n = 31)	Std Deviation	No (n = 81)	Std Deviation	Means difference	t	Sig. (2 tailed)
	1.076	0.43913	1.1571	0.46960	−0.08097	−0.831	0.408
Hard to reach district (DISTACCESS)	Yes (n = 25)	Std Deviation	No (n = 87)	Std Deviation	Means difference	t	Sig. (2 tailed)
	1.2596	0.55307	1.0988	0.42769	0.16081	1.547	0.125
Availability of TA for Pharmaceutical Management (TA) PHCPEFacility	Yes (n = 45)	Std Deviation	No (n = 67)	Std Deviation	Means difference		Sig. (2 tailed)
	1.2297	0.48252	1.0709	0.43781	0.15882	1.806	0.074

### Pearson’s correlation analysis

We performed correlation analysis to determine the relationship between per capita pharmaceutical expenditure and the various continuous variables. The results are shown in Table 4.

**Table 4:** **Correlation coefficients of per capita pharmaceutical expenditure (′000) according to levels of dichotomous variables**

**Variables**	**PHCPECapita**
Total Population: POPTOT	−.343**
Percentage Female: PERCFEM	−0.061
Percent rural population below poverty line: RURALPOV	−.282**
Immunization Coverage: DPT3COVER	0.038
Per Capita OPD utilization: OPDCAPITA	.498**
Access to Safe drinking Water: ACCESSWATER	−.264**
Latrine Coverage in Households:LATCOVERAGE	0.092
Human Development Index: HDI	0.018
Human Poverty Index: HPI	0.154
Labour Absorption Rate: LABOURABSRATE	0.113
Urbanisation Rate: URBANISATION	−0.145
Literacy Rate-Total: LITRATETotal	0.024
Literacy Rate-Female LITRATEFemale	0.061
Literacy Rate-Male: LITRATEMale	0.081
Number of Government Health facilities: HFGOVTOT	0.115
Number of hospitals in district:HOSPTOT	−0.129
Number of NGO health facilities: HFNGO	−0.055
Percentage of facilities that are HC II: PERCHCII	0.163
Percentage of facilities that are HC III: PERCHCIII	−.191*
Percentage of facilities that are HC IV: PERCHCIV	0.046
Percentage of Approved posts filled: STAFFSTRENGTH	−0.07
Percentage of population within 5 km to a Health Facility: ACCESS	0.107

*Correlation is significant at the 0.05 level (2-tailed).

**Correlation is significant at the 0.01 level (2-tailed).

There is a significant correlation between per capita pharmaceutical expenditure and total district population, rural poverty, access to drinking water and outpatient department (OPD) per capita utilisation (P < 0.01). The percentage of health facilities in the district that are HC III is also significantly correlated with per capita pharmaceutical expenditure (P < 0.05). Apart from OPD per capita utilisation which has a relatively strong correlation with per capita pharmaceutical expenditure (r = 0.498), all the other significant factors have a weak correlatation with per capita pharmaceutical expenditure (r < 0.5).

### Multivariable analysis

Using stepwise multiple linear regression analysis, we estimated various specifications for district per capita primary health care pharmaceutical expenditure (PHCPECapita). The results are shown in Table 5. The selected base model (Model 1) explains about 58% of the variation in per capita primary health care pharmaceutical expenditure between districts (Adjusted R^2^ = 0.578). The correlation coefficients between the variables included in the model were lower than 0.5 ruling out the possibility of multicollinearity.

**Table 5:** **Regression models for per capita primary health care pharmaceutical expenditure (′000)**

**Dependent variable: primary pharmaceutical expenditure per capita (PHCPECapita)**						
	**Base model (Model 1)**		**Reduced model (Model 2)**		**Further reduced model (Model 3)**	
**Explanatory variables**	**Coefficient**	**t-value**	**Coefficient**	**t-value**	**Coefficient**	**t-value**
Constant	−0.133	−0.309	0.881	5.366^*^	0.827	4.929^*^
OPDCAPITA	0.230	3.834^*^	0.241	3.949^*^	0.230	3.674^*^
POPTOT	−0.00000147	−5.644^*^	−0.00000154	−5.805^*^	−0.00000163	−6.029^*^
HFGOVTOT	0.014	4.717^*^	0.016	5.116^*^	0.016	5.162^*^
RURALPOV	−0.009	−4.908^*^	−0.010	−5.636^*^	−0.009	−4.976^*^
HPI	0.023	3.958^*^	0.013	2.931^*^	0.016	3.430*
DISTACCESS	0.243	2.974^*^	0.219	2.642	-	-
LITRATEMale	0.010	2.524	-	-	-	-
R^2^	0.606		0.580		0.550	
Adjusted R^2^	0.578		0.554		0.528	
F	21.535		22.753		24.448	

*P<0.01.

Apart from the constant and the variable related to male literacy in the district (LITRATEMale), all the other variables in the base model are significant (P < 0.01). Two variables in the model, namely the district total population (POPTOT) and percentage of district rural population below poverty line in 2005 (RURALPOV) have a negative coefficient indicating that an increase in these variables results in a decrease in per capita pharmaceutical expenditure. For example, a 1% increase in the percentage of the district rural population below poverty line in 2005, leads to 9 UGX decrease in per capita pharmaceutical expenditure all other factors remaining constant. The coefficients for the rest of the variables in the model are positive indicating that an increase in these variables results in an increase in per capita pharmaceutical expenditure. For example, a 0.1 increase in district OPD per capita attendance leads to a 23 UGX increase in per capita primary health care pharmaceutical expenditure all other factors remaining constant.

To verify that the results of the base model (Model 1) are robust to a different functional form, we performed a regression analysis based on the natural logarithm of the per capita pharmaceutical expenditure, using the same explanatory variables. The model is still significant overall (p < 0.01) and the predictive ability of the model does not change significantly (Adjusted R^2^ = 0.551) indicating that the new model explains about 55% of the variation in per capita primary health care pharmaceutical expenditure. The signs of the coefficients of the explanatory variables remain unchanged; however Human Poverty Index (HPI) and whether a district is categorised by MOH as hard to reach or not (DISTACCESS) are no longer significant at 1% level (p > 0.01) but still significant at 5% level (p < 0.05).

Finally, we developed various iterations of the initial model to enable us select a final allocation model. The aim was to identify a more parsimonious model without significant loss in explanatory ability. The results are shown in Table 5 (Model 2 and Model 3).

## Discussion

This study aimed at identifying which factors to consider in allocating primary health care pharmaceutical budgets to districts in Uganda. One possible approach would have been to specify a different equation for each way of expressing pharmaceutical expenditure (e.g. expenditure per facility, expenditure per patient visit or expenditure per health provider etc.) [6,7]. We took the more conservative approach of choosing just one way of expressing pharmaceutical expenditure (expenditure per capita) and then went ahead to estimate the regression equation like has been done in some studies [3-5]. The study uses past pharmaceutical procurement expenditure data to identify variables explaining primary health care pharmaceutical expenditure. This is in contrast to other studies that have used diagnosis data, pharmacy claims data and individual patient morbidity data [7,11-15]. Such data is not readily available in the Ugandan context.

The final model (Model 3) was selected because it is parsimonious compared to other models without significant loss in explanatory ability. The overall model and the variables included in the model are all significant (P < 0.01). This model explains about 53% of the current variation in pharmaceutical expenditure among districts. The variables included in this model are: OPD capita attendance, total district population, total number of government health facilities in the district, percentage of rural population below poverty line 2005 and the Human poverty index. These variables can be used as corrective variables in the formula currently being used by the government of Uganda to allocate primary health care pharmaceutical budgets to the various districts.

The outpatient department attendance per capita (OPDCAPITA) variable in the model is a direct reflection of demand for health care and therefore need. The expenditure generated from this demand is geared towards meeting the expressed need. The higher the demand the higher the expenditure. This calls for a higher budget allocation. This is supported by the positive coefficient of this variable in the model.

The total district population variable in the model (POPTOT) has a negative coefficient indicating that in the current allocation, increase in the total district population results in a decrease in primary health care per capita pharmaceutical expenditure. This is surprising as one would expect that increase in the covered population should lead to an increase in pharmaceutical expenditure due to increased utilisation of health services. The negative coefficient observed for this variable in our study may simply represent economies of scale in service provision as the number of people in the district increases. Alternatively, it may be a result of a distortion caused by Uganda’s population structure where only 2% and 20% of the population is aged above 65 years and below 5 years respectively [16]. These groups, especially the elderly (aged 65+) are associated with high pharmaceutical expenditure per capita [17,18]. If a large proportion of the population consists of these age groups, an increase in population would be expected to lead to an increase in pharmaceutical expenditure leading to a positive coefficient for the total district population variable. This is not the case in Uganda.

The selected model includes two socioeconomic variables which are the percentage of the district rural population below the poverty line 2005 (RURALPOV) and the Human Poverty Index (HPI). The relationship between socioeconomic status and health is one of the most robust and well documented findings in social science. However, the reasons for the relationship are less clear since plausible causal mechanisms run in both directions [19,20]. For example, one would expect that the higher the percentage of rural poor living below the poverty line, the higher the incident of diseases and hence the higher the observed pharmaceutical expenditure, justifying a higher budget allocation. In such a situation, one would expect the variable RURALPOV to have a positive coefficient, contrary to what is observed in this study. It is also possible that given their poverty status, the poor may not be able to access health care hence leading to low expenditure in an area where the poor are the majority [21]. Such a scenario would lead to the RURALPOV variable having a negative coefficient as observed in this study. However, for prospective need-based allocation formula for pharmaceutical budgets in Uganda, it is proposed that the percentage of rural population below poverty line 2005 (RURALPOV) variable should be removed because its negative coefficient in the current model represents an inequity factor in the present allocation system. Additionally, the 2005 data used in this data is outdated and may not be reflective of current circumstances.

The HPI measures deprivations in four dimensions: a long and healthy life-defined by vulnerability to death at a relatively early age- as measured by the probability at birth of not surviving to age 40; knowledge- defined by exclusion from the world of reading and communications- as measured by the percentage of adults (aged 16–65) lacking functional literacy skills; a decent standard of living, as measured by the percentage of people living below the income poverty line (50 per cent of the median adjusted household disposable income); and social exclusion as measured by the rate of long-term unemployment (12 months or more) [22]. The closer the index is to 0, the better, indicating the absence of human poverty; while the closer it is to 100, the more deprived the population is. The selected model suggests that more deprived districts should be given a higher budget allocation since one would expect a more deprived population to have higher health needs and hence higher pharmaceutical expenditure.

Considering the variables related to the supply side of healthcare, the total number of government health facilities in the district (HFGOVTOT) is a variable in the model to compensate districts for costs that exist outside the scope of measures of health need alone and should be included in the allocation formula. A high number of health facilities in the district is expected to result into increased utilisation of health services and higher pharmaceutical expenditure. However, it does not necessarily mean that the increased utilisation and expenditure is due to actual health need.

One variable which does not appear in the model that we finally select but which is worth considering is the variable related to whether a district is considered by MOH to be a hard to reach district or not (DISTACCESS). In the base model (Model 1) the variable is significant and has a positive coefficient. This suggests that districts that are characterized by MOH as hard to reach have a higher expenditure and should be allocated higher primary health care pharmaceutical budgets than other districts. MOH characterizes districts as hard to reach based on geography, among other factors. Geography can play an important role in influencing both individual health status and access to health services [23]. Allocation formulae offer a means to balance geographic disparities although the process is fraught with the difficulty of differentiating legitimate factors which reflect genuine variation in need from spurious, supplier induced discrepancies in expenditure [24]. A test of the null hypothesis for no difference in per capita primary health care pharmaceutical expenditure between districts characterized as hard to reach or not was not rejected (Table 3). As such, this variable can safely be omitted from any needs-based allocation formula.

The results of this study are partly similar to other studies that have found health services utilization (OPD attendance) covered population size (district population), location and health system organizational factors to be predictors of pharmaceutical expenditure [3-8]. However, unlike in this study where deprivation as measured by HPI was found to be important in predicting pharmaceutical expenditure, earlier studies in England have found deprivation (as measured by the Jarman Index) not to be important [9,10]. One explanation for this difference in findings could be the way the two indices are measured.

A key strength of this study is that it focuses on the interaction of need and demand, supply and health system organisation factors as variables explaining current primary health care pharmaceutical expenditure. Most prior research has mostly restricted its focus on need and demand factors (e.g. demographic and health status factors) with little or no examination of the interaction of demand and need factors with supply and system organisation factors [25-28].

Findings from this study could have important implications for the government of Uganda policy regarding primary health care pharmaceutical budget allocation to districts in Uganda. Based on the results of the study and the above discussion, it is recommended that for a prospective needs-based allocation of pharmaceutical budget to districts in Uganda, the following factors should be considered: OPD capita attendance, total district population, total number of government health facilities in the district; and the Human poverty index. This would be an improvement to the current formula which emphasizes just need factors (district population, mortality and live births indicators). This proposed formula considers social economic factors (human poverty index) as contributing to health need. And by including a variable related to the supply of health services in the district (total number of government health facilities), the formula tries to compensate districts for costs that exist outside the scope of measures of health need alone. However, being a utilisation driven formula, use of this formula has the risk of reinforcing any disparities in districts where there is systematic underutilisation of health services relative to health needs.

The proposed allocation is based on historical pharmaceutical expenditure data. Hence, it does not necessarily imply that the proposed allocation is efficient or equitable [29]. Although the primary rationale underlying needs-based formulae like the one proposed here is the accurate prediction of health care expenditure, the ‘fair’ distribution of resources appeals to a concern for vertical equity–that those with the greatest need should receive the greatest share of resources. Utilisation driven formulae like the one proposed here act to promote equality of access based on demand. However, they risk reinforcing health disparities in groups that systematically under-utilise health services relative to their health needs [30]. Since ‘unmet need’ is concealed by prevailing utilization patterns, the implication is that formulae must engage in some form of normative comparison between sub-populations if equity of health outcomes is to be achieved. A study to determine how equitable the current health service utilisation is would be useful in adjusting the proposed formula for unmet need.

## Limitations

The findings of this study could have been influenced by the study limitations. Some of the data for the explanatory variables was based on past national surveys that have not been updated. For example, the Human Poverty Index data used is based on the national survey conducted in 2007, and the rural poverty data used is from 2005. The assumption that these indicators have remained constant over the period in all districts of the country may not be entirely true. Any changes that have happened in these variables may result in either under or over estimation of the various parameters of the models due to inaccurate measurement of the variable. Also, through re-districting, many new districts have been created over the period by breaking up large districts into smaller ones. Data for new districts was missing for variables obtained from national surveys conducted before the districts were created. Gaps in data were filled by allocating the same variable value to a new district as the parent district. Whereas this was the best approach to fill gaps in the circumstances, it assumes homogeneity among all counties in the district, which may not necessarily be true.

The study did not take into account centralized pharmaceutical budget lines which cover pharmaceuticals for Malaria, HIV/AIDS, Family Planning and Tuberculosis. These “program” medicines are mainly funded by donors and more funds are used for their procurement compared to the essential medicines and health supplies considered in the study. It is estimated that 60% of health commodity financing in Uganda is donor dependent and focused on the program commodities which account for a large portion of the total pharmaceutical expenditure in each district [31]. Specifically, ACTs are one of the most widely prescribed medicines since Malaria is the leading cause of OPD attendance in health facilities [1]. However, spending on ACTs was not included in the study and this may have affected the results. Also, the results of this study may be subject to omitted variable bias due to the fact that data on district disease prevalence was not included as one of the study variables. Observed differences in expenditure between districts could be explained by differences in needs caused by differences in disease burden.

The value of pharmaceuticals procured by districts from NMS was used as proxy for pharmaceutical expenditure. This assumes that all the pharmaceuticals procured during the financial year were dispensed and that the facility started with no stock at the beginning of the financial year. Although high stock out rates have been reported in the public sector health facilities [32], this assumption is unlikely to be true since health facilities maintain some buffer stock for a number of commodities as per the national inventory management guidelines. Using actual dispensing/pharmacy data from health facilities would have been a better reflection of actual pharmaceutical expenditure.

Despite its limitations, the study proposes a simple, straight forward and parsimonious model for improving the prospective needs-based allocation of primary health pharmaceutical budgets to districts in Uganda. The model is based on readily available data and hence should be easy to apply. The model includes population factors related to health need; and by including a variable related to the supply and organisation of health services in the district the formula tries to compensate districts for costs that exist outside the scope of measures of health need alone.

## Conclusions

Based on the results of this study, proposed variables to consider in allocating prospective primary health care pharmaceutical budgets to districts in Uganda are: district outpatient department attendance per capita, total district population, total number of government health facilities in the district and the district human poverty index. As a way of validating the proposed budget allocation model, a comparison of trial pharmaceutical budget allocation based on these variables and actual budget spending for the various districts would be useful.

## Abbreviations

ACTs: Artemesinin based combination therapies
ARVs: Anti retrovirals
EMHS: Essential medicines and health supplies
FHSAs: Family health service areas
FY: Financial year
GOU: Government of Uganda
GPs: General practitioners
HC: Health centre
HPI: Human poverty index
MOH: Ministry of Health
NMS: National Medical Stores
OPD: Out patient department
PHC: Primary health care
UGX: Uganda shillings
